# Community Psychiatry Care: An Urgent Need in Nigeria

**DOI:** 10.2147/JMDH.S309517

**Published:** 2021-05-20

**Authors:** Modupeoluwa Omotunde Soroye, Obinna O Oleribe, Simon D Taylor-Robinson

**Affiliations:** 1 School of Public Health, University of Port Harcourt, Port Harcourt, Rivers State, Nigeria; 2 Department of Preventive Dentistry, Faculty of Dentistry, University of Port Harcourt, Port Harcourt, Rivers State, Nigeria; 3 Klamath Tribal Health & Family Services, Klamath Falls, OR, 97601, USA; 4 Department of Surgery and Cancer, St Mary’s Hospital Campus, Imperial College London, London, W2 1NY, UK

**Keywords:** community psychiatry, Nigeria, depression, mental health

## Abstract

Nigeria’s mental health policy was formulated in 1991, but it did not make adequate provision for community-based psychiatric care. Since there are only seven government-owned psychiatry facilities in Nigeria and these are always overwhelmed, there is the need to overhaul the existing policy and emphasise the urgency of a shift from inpatient psychiatric mental healthcare towards a community-based multidisciplinary psychiatric healthcare system.

## Introduction

The burden of psychiatric disorders worldwide is huge and it does not spare low- and middle-income countries (LMIC), such as Nigeria, where the burden of disease is currently great.^1^ According to World Health Organization (WHO), one in every four Nigerians has mental illness.^1^

Globally, many people who experience mental illness are reluctant to seek help, because of the stigma associated with it.^2^ Although the spectre of stigmatisation negatively impacts seeking help, there is little information about the nature of the associated factors that govern the avoidance of medical treatment.^2^ Of note, it has been reported that unmet needs for mental health treatment are particularly commonplace in LMICs.^1^

We discuss the issues surrounding mental health stigma in Nigeria, which as Africa’s most populous country acts as a microcosm for the continent as a whole. We also discuss the need to shift psychiatric care from a hospital-based program to a multidisciplinary community-based system involving psychiatrists, psychologists, nurses, social workers and occupational therapists, with active support from community and religious elders, which is extremely important in an African context.

## Discussion

Nigeria, the seventh-largest country in the world, has Africa’s highest caseload of depression, and ranks 15th in the world for the frequency of suicide.^3^ There are less than 150 psychiatrists in the country of 200 million people, with fewer than 10% of mentally ill Nigerians having access to the care they need.^3^ By contrast, there are about 28,000 psychiatrists in the USA for a population of 330 million people and 12,300 psychiatrists in the United Kingdom for a population of 68 million people.^4^,^5^ These comparative figures from the Global North highlight the stark requirements in Nigeria for more creative solutions in the absence of trained psychiatrists.

It should be noted that a significant number of people with mental illness are not treated in many communities in Nigeria, due to the myths and stigma associated with it.^6–10^ In Nigeria, there are various beliefs surrounding schizophrenia and depression. It is a taboo in some quarters to even discuss them. Some view people with schizophrenia as witches or demons possessed. The condition is considered a spiritual attack and a punishment for evil-doing from God for illicit psychoactive substance use, among other things.^11^,^12^ Some families even go so far as not allowing their relatives to marry into families with a history of mental illness. Others believe that there is no need for treatment, as the mentally ill can “snap out of it” if they try hard enough.^11^

Negative views about the mentally ill lead to unacceptable societal attitudes. Studies reported the same discriminatory attitudes among all cadres of people, including healthcare workers and even clergymen.^11–23^ There is the need for a shift from inpatient psychiatric mental health care towards a community-based care.

If community psychiatry is encouraged in Nigeria, it should focus on the detection, prevention, early treatment and rehabilitation of patients with emotional disorders, as they develop in the community, rather than waiting until hospital admission is required.^24^ This approach should be multi-disciplinary in order to succeed. It places emphasis on social-interpersonal environmental factors that contribute to mental illness. By engaging with community support through social mechanisms, such as the church or mosque, community psychiatric initiatives allow social acceptance at a grassroots level. Thus, treating mental illness in the community should improve the public attitude towards the mentally ill in Nigeria and lessen the devastating effect of caring for a mentally sick person by family members. More so, it should help correct the stigma associated with mental illness and help educate the community as regards the falsehood of the myths associated with it. Of note, it has been reported that psychiatric care in LMICs could also be improved by training primary healthcare workers to give mental health education to the communities they serve.^25–27^

Furthermore, community-based treatment makes it possible for people with mental disorders to maintain family relationships, friendships and jobs, while receiving treatment and facilitating early treatment and rehabilitation. This community-based approach also allows for continuity of care, increased adherence to treatment, better protection of human rights, and prevention of stigma.^28^ A reform of the mental health law that is in keeping with international standards is urgently needed to drive change.^28^

This is not to say that there are not excellent private-sector initiatives being undertaken currently in Nigeria. An example of this is “Mentally Aware Nigeria” (MANI), which is a non-governmental organisation (NGO).^29^ It has become West Africa’s largest youth-focused mental health organisation. MANI has over 1500 youth volunteers across Nigeria who aim to destigmatize mental health. The focus is on educating the populous and providing an environment which facilitates the young to source mental healthcare without being fearful of discrimination.

However, there is a need for a policy that establishes community-based psychiatry across Nigeria. The 2006 WHO-AIMS report on mental health system in Nigeria made some apt comments. It reported that “there is considerable neglect of mental health issues in the country”.^30^ The existing Mental Health Policy document in Nigeria was formulated in 1991. Since its formulation, no revision has taken place and no formal assessment of how much it has been implemented has been conducted. It appears that no desk exists in the Governmental Ministries at any level for mental health issues and only 4% of Governmental expenditure on health is earmarked for mental health.^30^

The report also discovered other issues related to mental health, such as the non-availability of essential medicines at health centres, the non-availability of physicians to run primary healthcare centres and the lack of, or restrictions on the prescription of psychotropic medications. In addition, there are only a few NGOs, apart from MANI, involved in counselling with no housing or support groups available.^29^,^30^ Thus, there is the need to overhaul psychiatry care in Nigeria with special attention placed on community-based services.

## Recommendations

In developing a policy for community psychiatry in Nigeria, we make the following proposals:

1. Incorporate psychiatry care into primary healthcare services for assessment and short-term treatment of less severe and time-limited disorders (anxiety, mood swings and mild substance disorders), and ongoing healthcare for people with severe mental health illness and/or significant risk factors.
2. Create multi-disciplinary mental health teams, consisting of psychiatrists, community nurses, psychologists, pharmacists and other healthcare workers, such as social workers and occupational therapists.
3. Incentivise psychiatrists to rotate into the Primary Health Centres (PHCs).
4. Train specialised healthcare teams to identify early signs of depression, schizophrenia, anxiety and substance disorders. Healthcare teams comprising psychiatrists, psychologists and mental health nurses should move between the community (educating about and identifying mental illnesses) and PHCs.
5. Institute comprehensive anti-stigma programmes.
6. Provide continuous education and support for parents and family members of the mentally ill patients.
7. Establish community-based psychotherapy (individuals, couples, family, groups), including cognitive behavioural therapy (CBT).
8. Train, examine and certify psychiatric nurses, psychologists and pharmacists to manage the use of psychiatric drugs, in order to reduce the bottleneck caused by the extreme shortage of psychiatrists.
9. Ensure two-way referral of severe cases to the major psychiatry centres.
10. Establish a monitoring and evaluation team in each local region that serves as a monitoring and Governmental advisory committee.
11. Promote and popularize mental health knowledge through the use of social media platforms and collaboration with NGO initiatives, such as MANI.
12. Strengthen the capacity of remote and online mental health services to allow them to be used robustly and reliably.

## Conclusions

The passage into law of a policy on community-based psychiatry should make psychiatric care more accessible to most Nigerians. This will, in addition, reduce the stigma attached to mental health illness, improve the quality of life of those suffering from it and provide the much-needed support for sufferers and their family members.
